# Membrane metallo‐endopeptidase is dispensable for repair after nerve injury

**DOI:** 10.1002/glia.23680

**Published:** 2019-07-24

**Authors:** Ilaria Cervellini, Jorge Galino, Ning Zhu, Florence R. Fricker, Lu Bao, David L. H. Bennett

**Affiliations:** ^1^ The Nuffield Department of Clinical Neurosciences University of Oxford, John Radcliffe Hospital Oxford UK; ^2^ Boston Children's Hospital Harvard Medical School Boston Massachusetts

**Keywords:** MME, NRG1, regeneration, remyelination

## Abstract

Membrane metallo‐endopeptidase (MME), also known as neprilysin (NEP), has been of interest for its role in neurodegeneration and pain due to its ability to degrade β‐amyloid and substance‐P, respectively. In addition to its role in the central nervous system, MME has been reported to be expressed in the peripheral system, specifically in the inner and outer border of myelinating fibers, in the Schmidt‐Lantermann cleft and in the paranodes. Recently, mutations of this gene have been associated with Charcot‐Marie‐Tooth Type 2 (CMT2). Peripheral nerve morphometry in mice lacking MME previously showed minor abnormalities in aged animals in comparison to CMT2 patients. We found that MME expression was dysregulated after nerve injury in a Neuregulin‐1 dependent fashion. We therefore explored the hypothesis that MME may have a role in remyelination. In the naïve state in adulthood we did not find any impairment in myelination in MME KO mice. After nerve injury the morphological outcome in MME KO mice was indistinguishable from WT littermates in terms of axon regeneration and remyelination. We did not find any difference in functional motor recovery. There was a significant difference in sensory function, with MME KO mice starting to recover response to mechanical stimuli earlier than WT. The epidermal reinnnervation, however, was unchanged and this altered sensitivity may relate to its known function in cleaving the peptide substance‐P, known to sensitise nociceptors. In conclusion, although MME expression is dysregulated after nerve injury in a NRG1‐dependent manner this gene is dispensable for axon regeneration and remyelination after injury.

## 1 INTRODUCTION

Membrane metallo‐endopeptidase (MME) gene encodes a zinc‐dependent metalloprotease, a Type II transmembrane glycoprotein of 100 kDa with the catalytic site localized in the extracellular domain. MME is expressed on lymphoblasts of patients with acute lymphoblastic leukemia (Brown, Hogg, & Greaves, 1975; Greaves, Brown, Rapson, & Lister, 1975), in kidney, breast, intestine (Metzgar, Borowitz, Jones, & Dowell, 1981), in glioma and melanoma cells in vitro (Carrel, de Tribolet, & Mach, 1982) and in fibroblasts (Braun, Martin, Ledbetter, & Hansen, 1983). In the central nervous system (CNS) MME regulates the levels of neuropeptides and is the main β‐amyloid degrading enzyme (Pacheco‐Quinto, Eckman, & Eckman, 2016) (Pacheco‐Quinto et al., 2016; Turner, Isaac, & Coates, 2001).

In the peripheral nervous system (PNS) MME has also been shown to have an important role in the regulation of neuropeptide levels. MME deficiency has been linked with exacerbation of pain and neurogenic inflammation after nerve injury. MME knock‐out mice showed increased sensitivity to heat, cold, mechanic stimuli, and reduced motor activity after chronic constriction injury (CCI) on the sciatic nerve (Kramer, He, Lu, Birklein, & Sommer, 2009). This effect has been attributed to decreased neuropeptide catabolism due to the lack of MME neuropeptidase activity and indeed levels of substance P and endothelin‐1 were increased (Kramer et al., 2009). Cadoni et al. (1993) reported that MME was absent in the CNS myelin but expressed in the inner and outer border of myelinated fibers in the PNS, in the Schmidt‐Lantermann clefts and in the paranodal regions. Additional support of a possible association of MME with peripheral myelination came from a gene profiling study investigating the embryonic development and myelination of Schwann cells. MME expression was found to be upregulated in the sciatic nerve between E18 and P12 and down‐regulated after nerve cut (D'Antonio et al., 2006). To our knowledge, no further study has been conducted since then to investigate the role of MME in peripheral nerve myelination or re‐myelination.

Interestingly, MME gene mutations have been of recent interest due to their association with late‐onset Charcot‐Marie‐Tooth Type 2 (CMT2; in which axonal pathology dominates). A recent clinical study in Japanese patients with CMT2 reported recessive mutations in the MME gene in 10 patients with adult onset axonal neuropathy and indicated those loss of function mutations as the most common cause of adult‐onset Autosomal‐Recessive CMT2 in Japanese patients (Higuchi et al., 2016).

A second study, identified heterozygous mutations in the MME gene as candidates for late‐onset autosomal‐dominant CMT2 disease in European and American patients (Auer‐Grumbach et al., 2016). The peripheral nerve abnormalities in aged mice lacking MME are subtle and much less marked than those seen in CMT2 patients. This could relate to the strength of the association between MME variants and the clinical phenotype or the limitations of the mouse in modelling late onset neuropathies. One further possibility is that MME is important in the repair response of the PNSs and in patients lacking MME they could accumulate deficits with aging.

In this study, we investigated the expression of MME during development and following nerve injury. We studied the effect of MME ablation on axon regeneration and in remyelination after injury and found that this endopeptidase is dispensable for axon regeneration and remyelination after nerve injury in rodents.

## 2 METHODS

### 2.1 Animals and surgery

All procedures were carried out in conformity to UK Home Office regulations and in line with the Animals Scientific Procedures Act 1986 in a licensed facility at the University of Oxford. Animals were housed in IVC cages in temperature and humidity controlled rooms.

C57BL/6 WT mice, bred in house, were used for qPCR analysis of gene expression during development and as control mice. Conditional NRG1 KO mice (conNRG1) and their control littermate were used to confirm MME gene expression 12 days after injury in absence and presence of NRG1 expression respectively (as described in [Fricker et al., 2013]). These mice were obtained by crossing CAG‐Cre‐ER™ mice (JAX® mice 004682), in which a tamoxifen inducible form of Cre recombinase is ubiquitously expressed, with Nrg1^fl/fl^ mice (Hayashi & McMahon, 2002). Both colonies were on a C57BL/6 background.

MME KO mice were originally generated at Harvard Children Hospital as described in Bao (1996), Lu et al. (1995)). This mouse model has previously been validated and shown to lack the relevant enzymatic activity (Bao, 1996; Lu et al., 1995). MME KO mice were compared with WT mice and equal number of animals of each gender was used in the experimental groups.

Sciatic nerve injury was performed in 8 weeks old MME KO mice and controls under sterile conditions. ConNRG1 mice and their control littermates were dosed by oral gavage with tamoxifen (Sigma T5648, 0.25 mg/g body weight in corn oil) when 8 week old and injured 1 month later. All mice subjected to injury were anaesthetized and the left sciatic nerve was exposed. Keeping the lesion site constant for all animals, the sciatic nerve was crushed twice (30 s each time) using fine forceps. Analgesic drugs were given postsurgery and the mice monitored regularly. Mice were sacrificed at different time points (as described later) and tissues taken from the injured and uninjured side for following examinations.

### 2.2 Behavioral tests

All behavioral testing were carried out with the observer blind to the genotype of the animals.

*Sciatic functional index*. Mice were trained to walk along a 1 m long suspended bar into a dark box. The bar was covered with a paper of the same dimension and the hind paws of the mice were inked with stamp ink. Paw prints were then analyzed manually. Both uninjured and injured paw prints were measured as follow: toe spread as distance between first and fifth toe, intermediate toe spread as distance between second and fourth toe, and print length measured between the end of the third toe and the bottom of the paw. These measurements were used to calculate the sciatic function index (Bain, Mackinnon, & Hunter, 1989; Inserra, Bloch, & Terris, 1998) a measure of motor functional recovery after injury.

*Beam test*. Mice were recorded with a camera while walking along a 1 m wooden beam. Videos were then analyzed and the percentage of incorrect steps (slips and hops) was calculated against the total number of steps for each mouse. This test is a measure of proprioception in mice.

*Toe spreading reflex*. The aim of this test is to assess motor‐muscular function as result of small muscle reinnervation in the foot. The tail was gently lifted in response to which the mice spread their legs and digits. Toe spreading reflex was graded from 0 to 2:0 no spreading; 1 intermediate spreading; 2 normal spreading.

*Pinprick test*. This test assess the reinnervation of the skin by sensory afferents of the sciatic nerve. A fine insect needle was used to apply a pinprick stimulus in specific parts of paw and toes in the injured side. Score was given from 0 to 2:0 nonreflex withdrawal response, 1 mild reflex, and 2 normal reflex.

### 2.3 RNA isolation, cDNA transcription, and quantitative real time PCR

Fresh sciatic nerves were collected, immediately frozen in liquid nitrogen and stored at −80C. Tissues were then homogenised in TriPure (Roche) with a handheld homogeniser (Cole‐Parmer), treated with chloroform and purified with a High Pure Tissue Kit (Roche). RNA was eluted in RNase free water and cDNA was synthesised using a Transcriptor reverse transcriptase (Roche), random hexamers (Invitrogen) and dNTPs (Roche). cDNA amplification (5 ng) was done using the LighCycler 480 SYBR Green Master (Roche) and primers (0.5 μM). White 384 well plates (Roche) were run for 45 cycles in a LC 480 II system (Roche). MME primers were designed using Primer‐BLAST (https://www.ncbi.nlm.nih.gov/tools/primer‐blast/) and validated before the experiment. The MME primer used for KO mice was specifically designed in order to include exons 12 and 13 that were modified to obtain the KO mouse model: CAGCCTCAGCCGAAACTACA (forward primer) and ACATAAAGCCTCCCCACAGC (reverse primer). Gene expression was normalized against three reference genes (GAPDH, HPRT1, and 18S) and calculated by the ΔΔCt method.

### 2.4 Western blotting

Sciatic nerve tissues were homogenized in RIPA lysis buffer plus protease inhibitor cocktail (Roche). Lysates were keep rotating for 90 min at 4 °C and then spun at 13,000 rpm for 15 min. The protein concentration of the supernatant was determined using a BCA Protein assay kit (Thermo Scientific). Proteins (15 μg) were loaded in 10% Mini‐PROTEAN® TGX™ Precast Gels (Biorad) and transferred to a Nitrocellulose membrane (Biorad), blocked in 10% milk in PBS 0.1% Tween. Primary antibody used were αGt MME (Life Technologies; 1:1,000) and αMs GAPDH (Abcam; 1:500). Secondary antibodies were anti‐goat (Abcam; 1:20,000) and anti‐mouse (GE Healthcare; 1:10,000) IgG horseradish peroxidase linked. ECL prime detection kit (GE Healthcare) was used to visualise immunoreactive bands on chemiluminescence film (GE Healthcare). Quantification was done using ImageJ software and expression was normalised against GAPDH loading control.

### 2.5 Immunofluorescence microscopy

Mice were perfused with 5 mL saline followed by 10 mL paraformaldehyde (PFA 4% in 0.1%M phosphate buffer).

Skin biopsies were taken from the hindpaw glabrous skin proximal to the last paw pad, postfixed in PFA 4% for 30 min then transferred to 20% sucrose overnight (4°C) and successively blocked in OCT embedding compound on dry ice and stored at −80°C. Transverse sections were cut on a cryostat at the thickness of 14 μm onto gelatin coated Super Frost Ultra Plus slides. C‐fibers in the epidermis were visualized by immunostaining with polyclonal rabbit PGP 9.5 (1:200, Zytomed). Secondary antibody used was anti‐rabbit Cy3 (1:1,000, Stratech Scientific). Epidermal fibers were counted at a ×63 magnification live on the microscope according to the rules set out by Lauria (2005), Lauria et al. (2005). Four sections per animal were counted with the analyst blind to genotype. The length of the epidermis was measured by ImageJ in images taken with ×20 magnification, and used to calculate the density of intra epidermal fibers (IENFD) as fibers per millimeter.

For nerve teasing staining the sciatic nerve was incubated with 0.5% Osmium tetroxide (TAAB) for 2 hr. Nerves were then washed with increased EtOH concentrations and stored in 50% glycerol and 50% of 70% EtOH. Fibers were teased with fine needles under a dissection microscope after perineurium removal. Slides were then mounted with Vectarshield mounting medium for fluorescence (VECTOR) and analyzed at the confocal microscope. Internodal distance was measured using ImageJ.

### 2.6 Electron microscopy

Sciatic nerves from naïve and injured mice (2 mm distal to the crush site) were postfixed with 4% PFA and 3% glutaraldehyde in 0.1 M phosphate buffer as previously described (Fricker et al., 2009). Nerves were treated with 1.5% osmium/0.2 M PB for 90 min, dehydrated and embedded in epoxy resin (TAAB embedding materials). Ultrathin sections (90 nm), obtained with a Diatome diamond knife on the Leica UC7 ultramicrotome, were mounted onto 100 mesh Formvar coated Cu grids. After a 5 min staining with lead citrate, pictures were taken with a FEI Tecnai T12 transmission electron microscope (TEM) at the Dunn School Bioimaging facility (University of Oxford). For axons and macrophages analysis, photographs were taken of random squares of the grid, covering about 20% of the total area of the cross section of the sciatic nerve. Counts were normalized for the area in mm^2^, after confirming that the nerve area was comparable between control and KO mice. Analysis was done using the AxioVision LE, release 4.2 software.

### 2.7 Experimental design and statistical analysis

All statistical analysis has been performed using the GraphPad Prism 6 software. Data are reported as means ± standard error (*SEM*) and the number of replicates is indicated in each figure legend. The level of significance was set at *p* < .05. The two‐tailed unpaired Student's *t*‐test was used to analyze differences between two groups. To assess statistical differences in behavior experiments with multiple time points, repeated measures two‐way AVOVA was performed with Sidak's multiple comparison tests.

## 3 RESULTS

### 3.1 MME expression is downregulated in conditional NRG1 mutant mice after nerve injury and upregulated during early development

**Figure 1:**
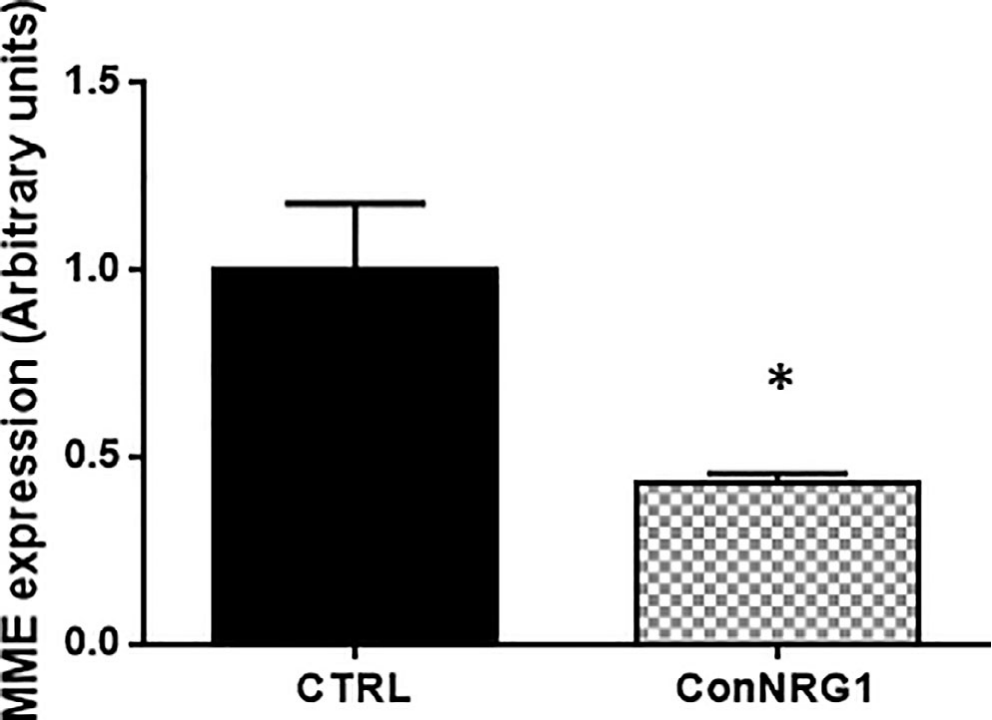
MME expression is decreased 12 days after nerve injury in mice lacking NRG1 compared to WT. qPCR analysis of MME expression in sciatic nerve of ConNRG1 mice and littermate controls at 12 days after nerve crush. Data are presented as mean ± *SEM* (*p < .05 student *t*‐test), *n* = 5

**Figure 2:**
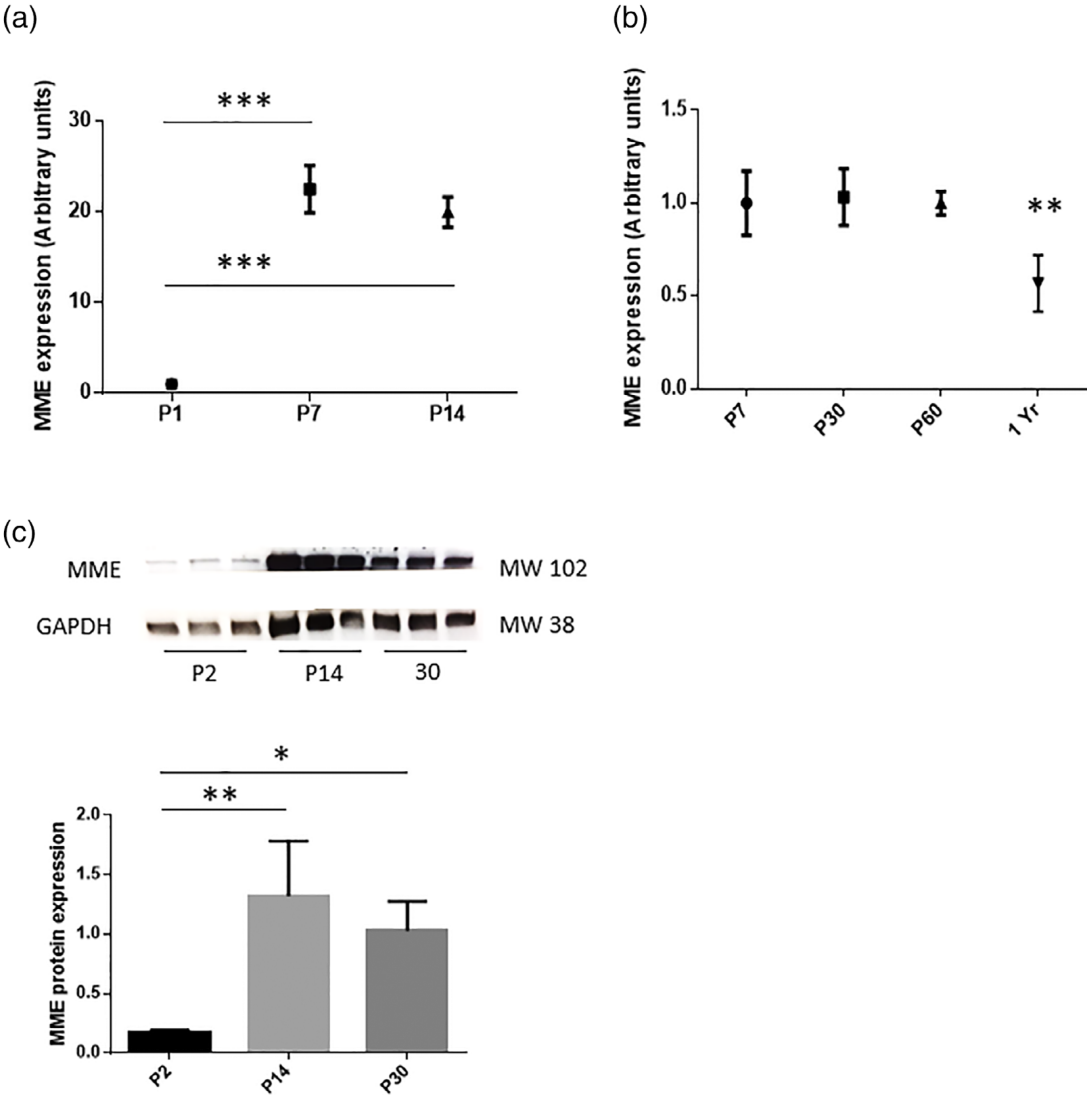
MME expression is increased during development in WT mice. (a) Sciatic nerves of P1, P7, and P14 WT mice were analyzed by qPCR for MME expression. Data are presented as mean ± *SEM* (***p < .001 one‐way ANOVA), n = 3. (b) P30, P60, and 1 year old sciatic nerves were compared to P7 sciatic nerves by qPCR for MME expression. Data are presented as mean ± *SEM* (**p < .005 one‐way ANOVA), *n* = 3–5. (c) Western blot analysis of P2, P14, and P30 sciatic nerves to show MME protein expression. GAPDH used as internal control. Data are presented as mean ± *SEM* (*p < .05; **p < .005 one‐way ANOVA), *n* = 3 [Color figure can be viewed at wileyonlinelibrary.com]

NRG1 is a growth factor required in the early recovery phase after nerve injury and determines the rate of remyelination (Fricker et al., 2011, 2013; Stassart et al., 2013). NRG1 regulates the expression of many myelin‐related genes following sciatic nerve crush and we previously identified them by performing a genome‐wide transcription profiling using Affymetrix Gene Chip Mouse Gene 1.0 ST Arrays (Fricker et al., 2013). As previously shown in Fricker paper mice lacking NRG1 (ConNRG1, treated with tamoxifen) and control littermate mice (Vh, Vehicle treated) were compared by a differential expression analysis at the naïve state (uninjured) and 10 and 28 days post sciatic nerve crush. As part of this analysis, we noted that MME expression was downregulated in control animals 10 days after injury and upregulated again at Day 28, when axon remyelination and regeneration are well established. The expression of MME was NRG1 dependent: MME expression was reduced in the absence of NRG1 at both Day 10 and 28 after injury with an FDR adjusted *p*‐value at Day 10 of *p* < .1 ((Fricker et al., 2013) supp_awt148_brain‐2012‐02167‐File017.docx). No difference was found between vehicle and tamoxifen treated mice in the uninjured sciatic nerve (Fricker et al., 2013) supp_awt148_brain‐2012‐02167‐File017.docx). We confirmed here the microarray results using qPCR analysis in ipsilateral sciatic nerves of control and conNRG1 mice 12 days after injury showing that MME expression was significantly reduced post injury in the absence of NRG1 (Figure 1). MME was previously reported to be expressed in myelinated fibers of the PNS (Cadoni et al., 1993) and to possibly have some role in myelination (D'Antonio et al., 2006). We also investigated its expression in naïve mice during development at P7 and P14, the time points in which myelinating Schwann cells are ensheathing the myelinating axons. MME gene expression was significantly increased at both of these time points (Figure 2a). At later time points of P30 and P60 there was no difference in MME gene expression compared to P7 showing that the gene expression remains stable during adulthood (Figure 2b). However, we found a significant decreased expression of MME in 1 year old mice (Figure 2b). Finally, we also detected a significant increase in protein expression from P2 to P14 with no further change in P30 old mice (Figure 2c).

In conclusion, our data indicate that MME is upregulated during development in the active phase of myelin formation.

In addition, this endopeptidase is downregulated in the early phase after nerve injury and still remains low expressed at a later time point when NRG1 expression is ablated.

### 3.2 MME ablation does not alter nerve morphometry in the naïve state

**Figure 3:**
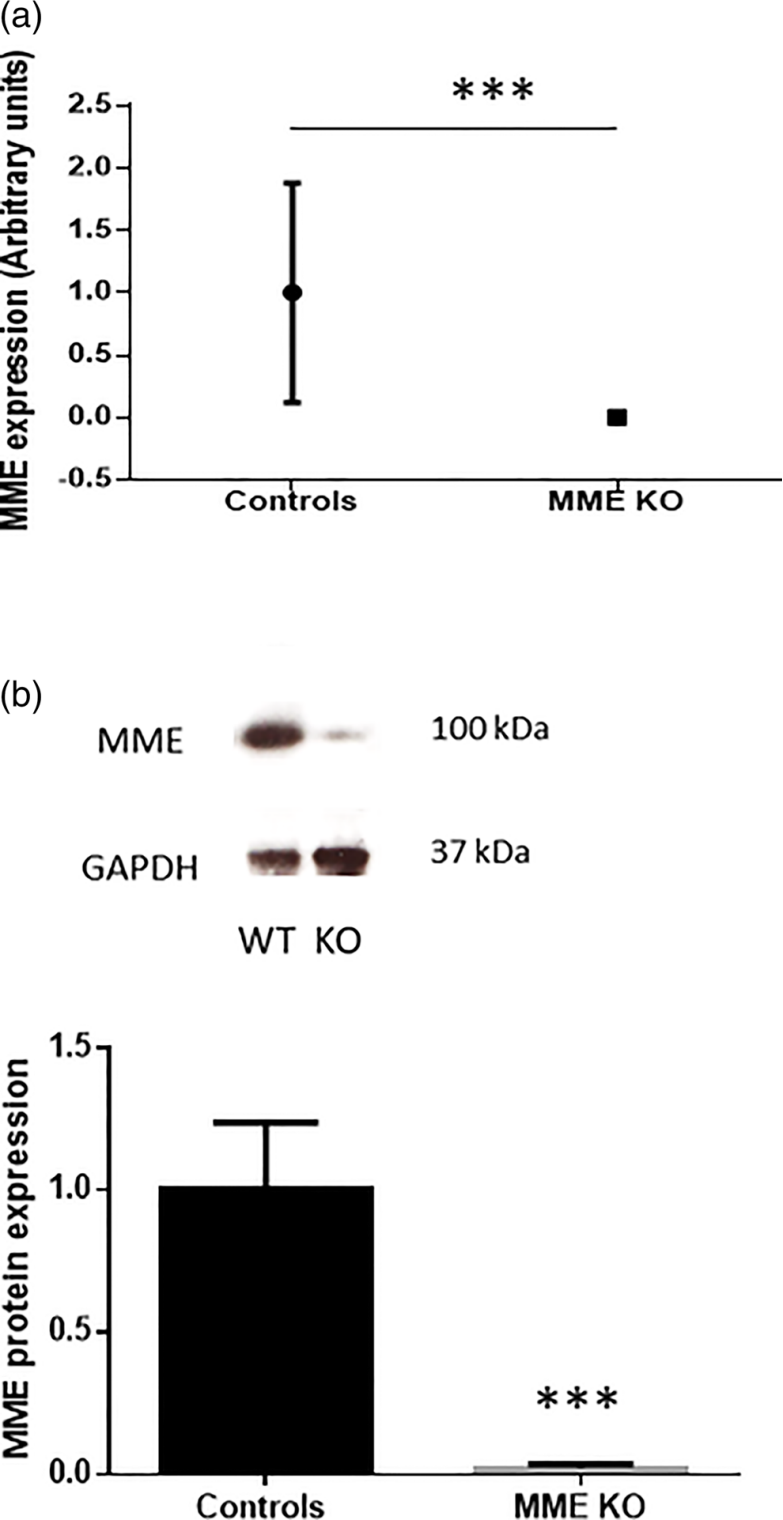
Validation of MME KO mice in the PNS. (a) qPCR analysis of sciatic nerves of controls and MME KO mice for MME gene expression. Data are presented as mean ± *SEM* (***p < .001 student *t*‐test), *n* = 5. (b) Western blot analysis of controls and MME KO sciatic nerves for MME protein expression. Data are presented as mean ± *SEM* (***p < .001 student *t*‐test), *n* = 4 [Color figure can be viewed at wileyonlinelibrary.com]

**Figure 4:**
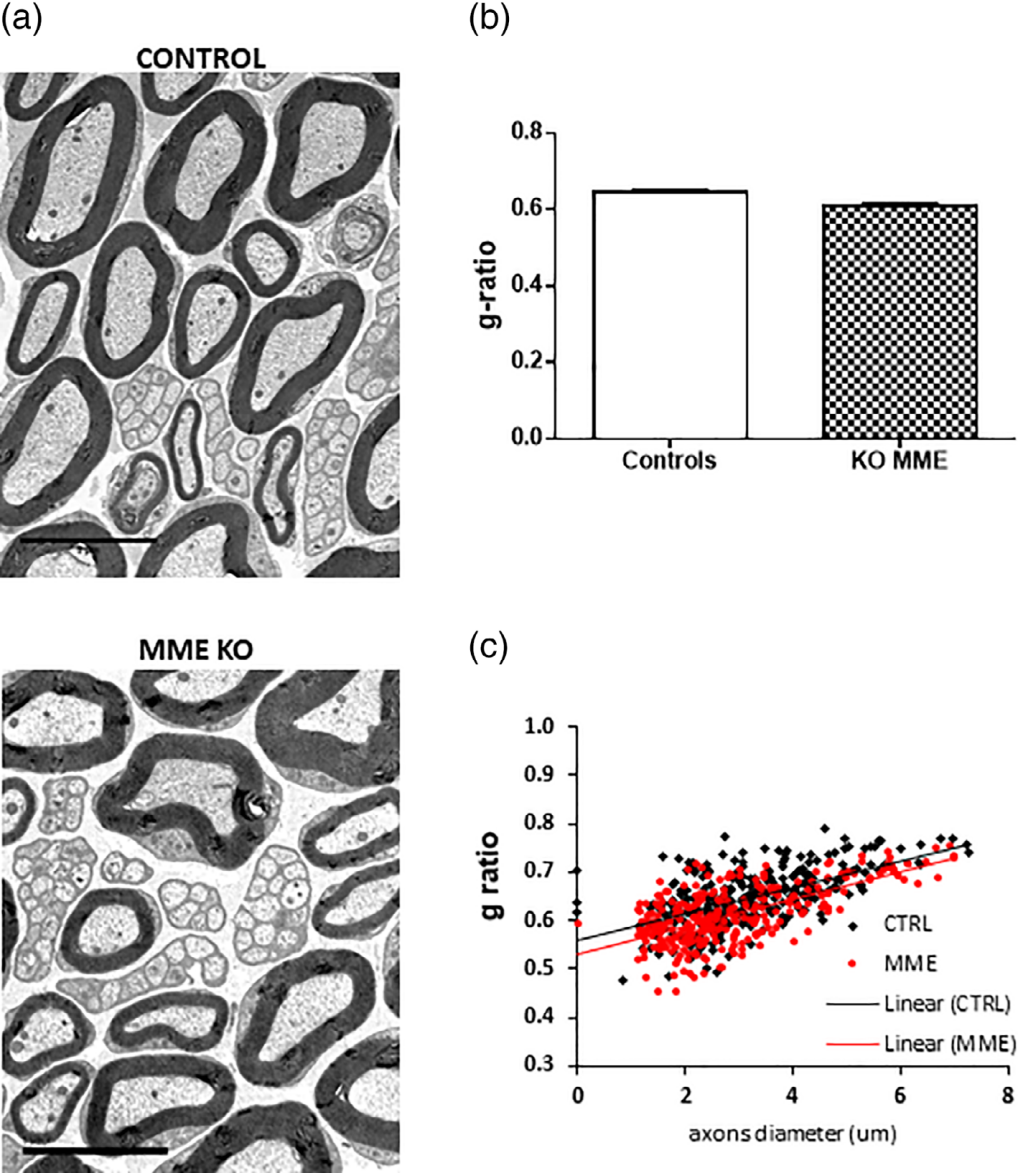
Ablation of MME does not affect myelination in adult mice. (a) Electron micrographs of transverse sections of WT and MME KO adult sciatic nerves in the naïve state. (b) g‐ratio quantification (no significance with student *t*‐test). The g‐ratio of individual fibers in relation to their axon diameter is plotted in c. Data are presented as mean ± *SEM*. Scale bar 5 μm, *n* = 3 [Color figure can be viewed at wileyonlinelibrary.com]

Following our previous findings of an increased MME gene expression at the time of early myelination events and taking advantage from the availability of a widely used MME KO mouse model, we investigated nerve morphometry in the naïve state. First, we wanted to confirm that MME gene expression was ablated in the sciatic nerves of MME KO mice. We found a significant decrease MME mRNA assessed by qPCR as reported in Figure 3a compared to control mice. In addition, MME protein expression was found to be markedly and significantly decreased in these KO mice (Figure 3b) confirming that this was a valid model to be used in our studies. Sciatic nerves from 1 month old WT and KO MME mice were analyzed by TEM. No morphological differences were found between the two groups as visible in Figure 4a and the quantification of the myelin thickness by g‐ratio measurement confirmed that (Figure 4b). Following a more detailed analysis of myelinated fibers, we did not found any difference also in the g‐ratio distribution per axon diameter (Figure 4c). As shown in Table 1, there was no difference between the two groups in the total axon number, in the number of myelinated and unmyelinated axons and in the c‐fibers distribution per Remak bundle. Macrophage count was also comparable between the two groups. All quantifications were normalized for the area in square millimeters since no difference was found in the nerve size between WT and KO mice.

**Table 1:** MME ablation does not affect nerve morphometry in adult mice

	Control	MME KO	*t*‐test
Total axon number/mm^2^	102405.1	105004.5	0.9
Myelinated axon number/mm^2^	28248.3	34311.7	0.2
Non myelinated axon number/mm^2^	74156.8	70692.8	0.8
c‐fibers/Remak bundle	13.8	13.5	0.9
Macrophage number/mm^2^	611.5	625.3	1.0

*Note*: No differences were found between adult MME KO and WT controls in the naïve state in relation to the total axon number, the number of myelinated and unmyelinated fibers and the distribution of C‐fibers per Remak bundle. Macrophage number was also comparable. Counts were normalised for area in square millimetres since no differences were reported in nerve size. *n* = 3–4 mice per group; two‐tailed unpaired *t* test.

### 3.3 MME ablation does not affect remyelination and regeneration after nerve injury

**Figure 5:**
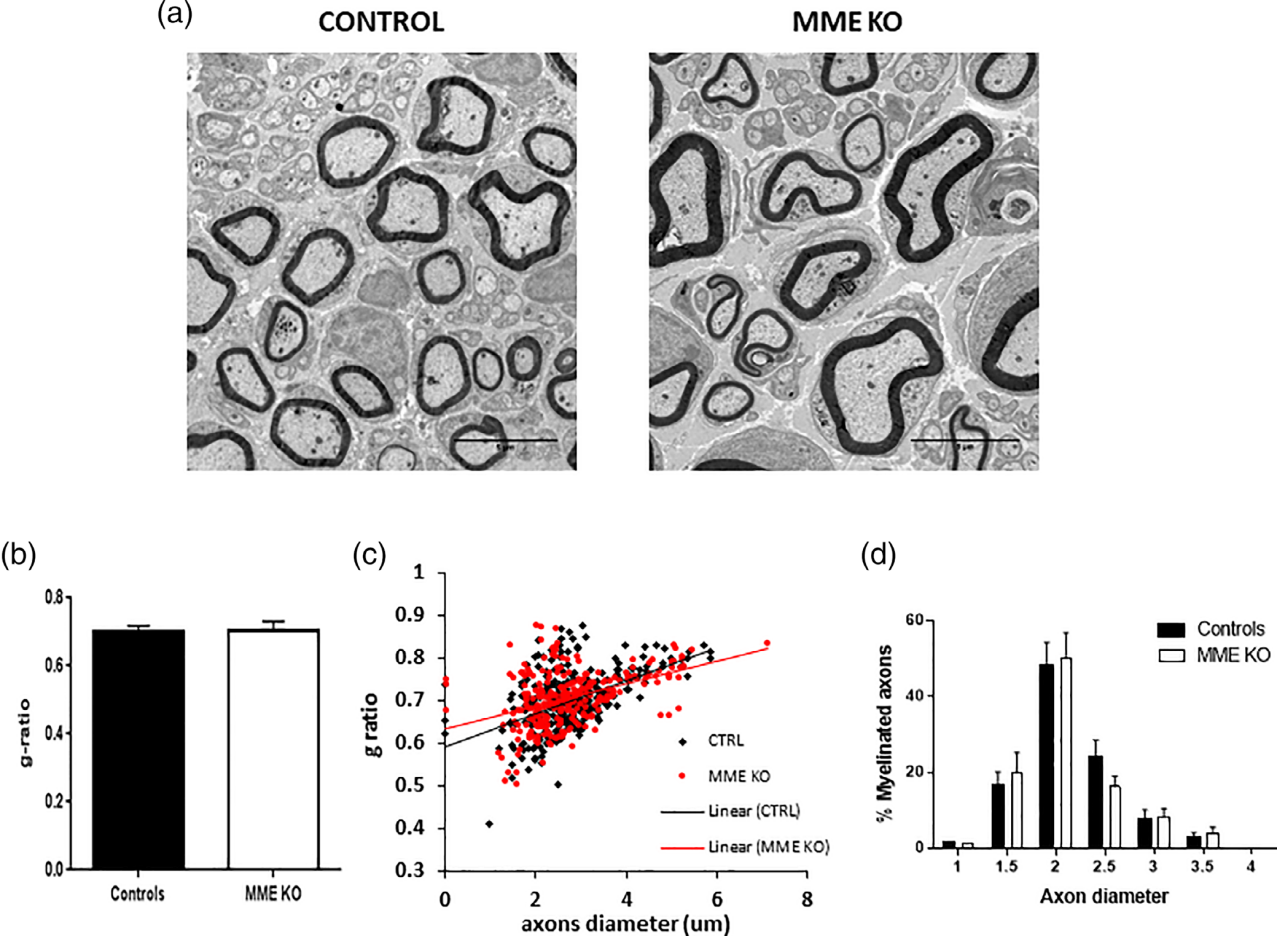
Ablation of MME does not affect myelin thickness nor myelinated axon distribution after nerve injury. (a) Electron micrographs of transverse ipsilateral sciatic nerve sections 1 month after injury. (b) g‐ratio analysis (no significance with student *t*‐test). (c) Scatter plot of g‐ratio versus axon diameter distribution. (d) Frequency distribution of myelinated axon diameter. Data are presented as mean ± *SEM*. Scale bar 5 μm, *n* = 3 [Color figure can be viewed at wileyonlinelibrary.com]

To assess the role of MME in nerve repair, we employed the model of sciatic nerve crush performed in adult WT and MME KO mice. One month following the injury, mice were sacrificed and the sciatic nerve was collected 2 mm distally from the crush site for TEM analysis. As shown in Figure 5a, we did not observe any difference in the morphology of regenerating sciatic nerves in the two groups as in both groups the axon density and remyelination were well advanced at 1 month postinjury. Myelin thickness as assessed by g‐ratio (Figure 5b) and its relationship to axon diameter (scatter plot in Figure 5c) were comparable between the groups. Percentage of myelinated fibers in relation to the axon diameter was also comparable (Figure 5d). We did not detect any difference in the number of myelinated and unmyelinated axons within the sciatic nerve or in the distribution of c‐fibers within the Remak bundles, as summarized in Table 2.

**Table 2:** MME ablation does not affect remyelination as showed by TEM analysis 1 month after sciatic nerve crush

	Control	MME KO	*t*‐test
Total axon number/mm^2^	143375	124917	0.54
Myelinated axon number/mm^2^	24188	22042	0.58
Non myelinated axon number/mm^2^	119188	102875	0.60
c‐fibers/Remak bundle	9	6	0.10

*Note*: Analysis of electron micrographs of transverse sciatic nerves at 1 month post ipsilateral nerve crush did not show any difference in axonal survival (total axon count) and in remyelination (as shown by the count of myelinated and unmyelinated fibers). Distribution of C‐fibers/Remak bundle was also comparable. *n* = 3–4 mice per group; two‐tailed unpaired *t* test.

**Figure 6:**
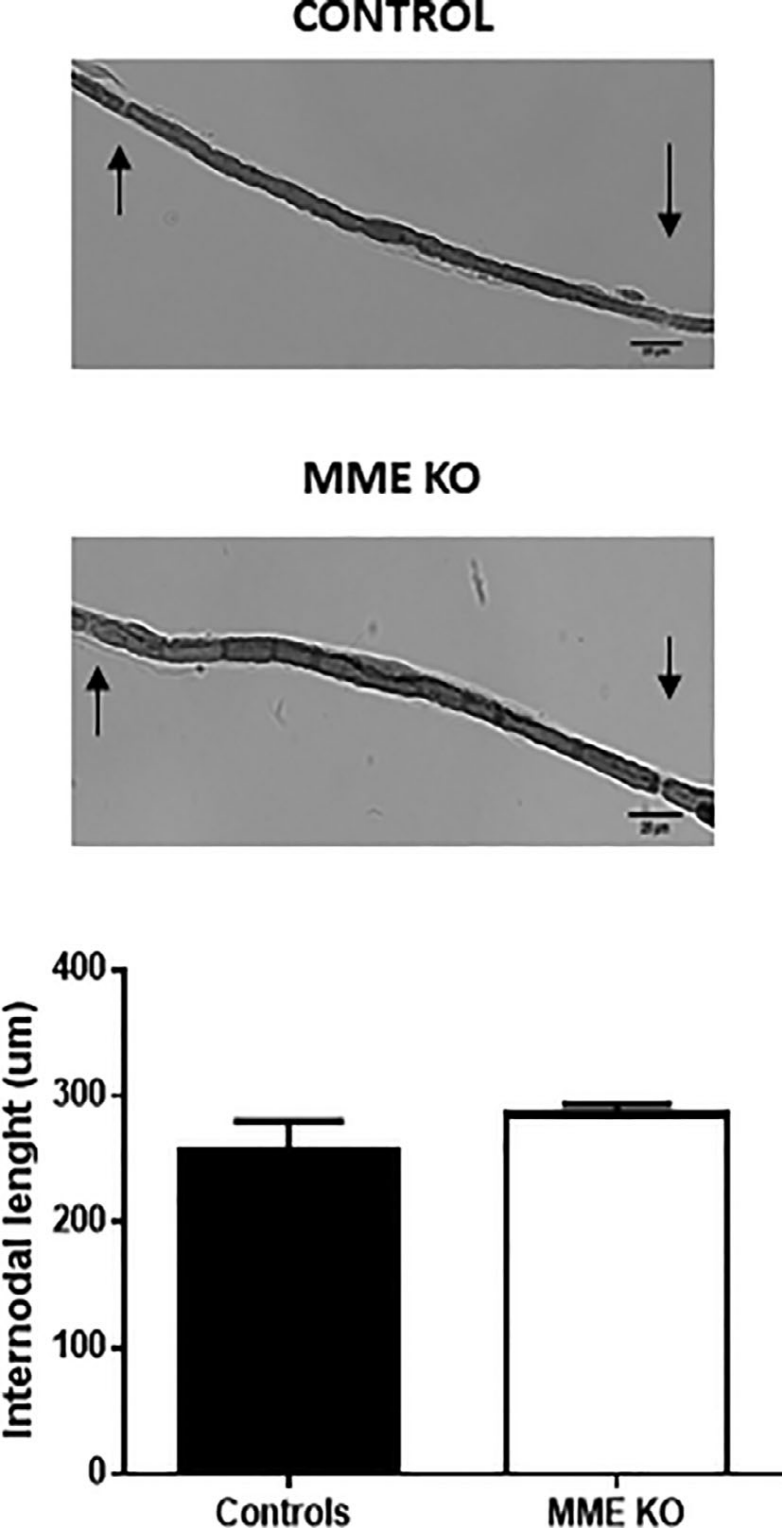
Schwann cell elongation after injury is comparable between MME KO and WT mice. Internodal distance measurement, as sign of Schwann cell elongation after injury, in teased sciatic nerve fibers. Scale bar 25 μm. For both analysis no differences were observed by two‐tailed unpaired *t*‐test. Data are presented as mean ± *SEM*, *n* = 3

Finally, we analyzed the effect of MME ablation in Schwann cell elongation after injury. We collected ipsilateral sciatic nerves at the end of the experiment and we measured the internodal distance in teased fibers. No differences were found in this quantification between the two groups (Figure 6).

We can therefore conclude that MME expression is dispensable for remyelination and axon regeneration after nerve injury.

### 3.4 MME is not required for functional motor recovery following peripheral nerve injury but does modulate response to noxious mechanical stimuli

**Figure 7:**
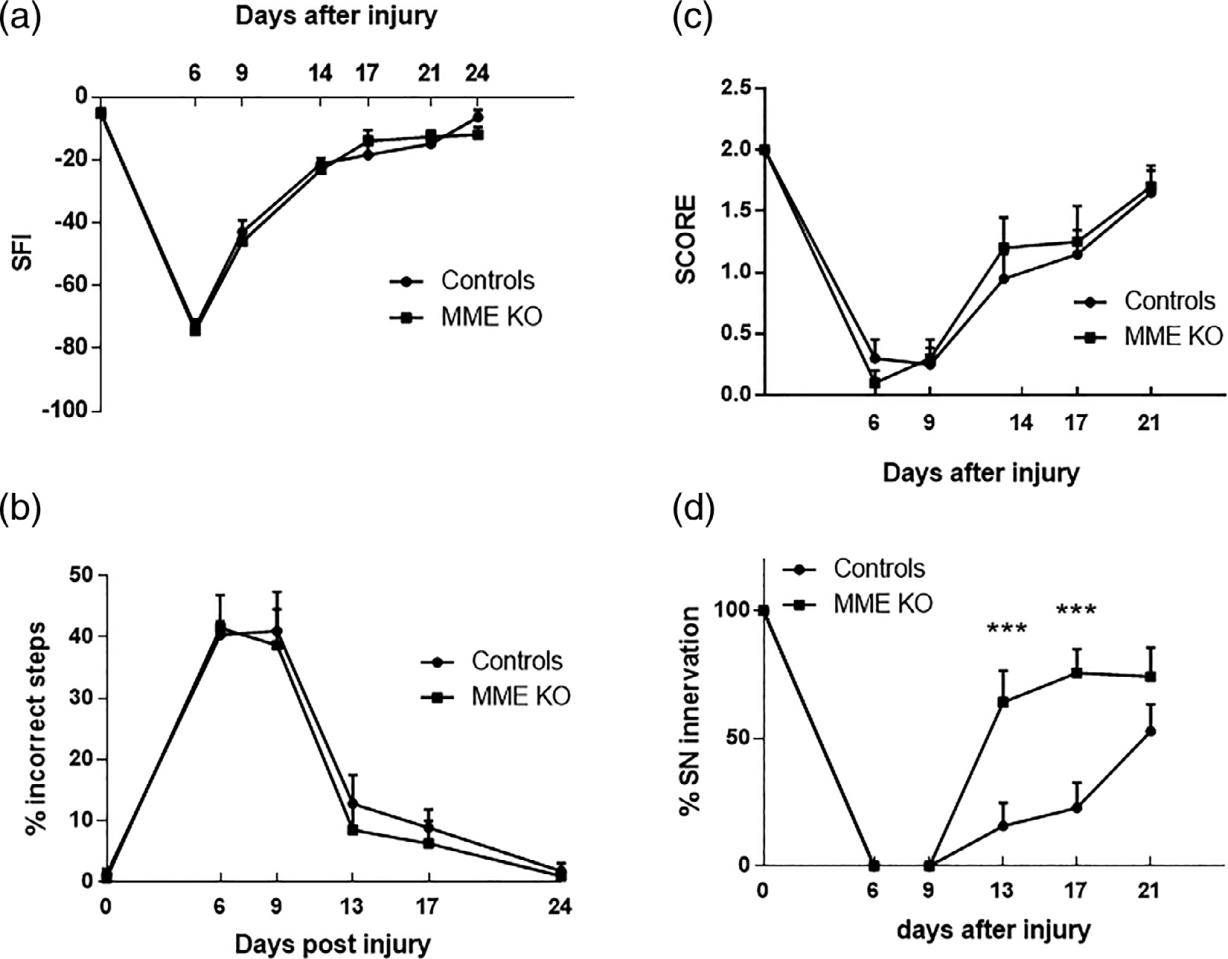
Functional outcomes in MME KO and WT mice 1 month after injury. (a) Sciatic functional index was used to assess gait and locomotion. (b) Beam test was used to assess balance and motor coordination after injury. (c) Motor function was measured by the toe spreading reflex test. None of these tests demonstrated a significant difference between MME KO and WT littermate controls. (d) Pinprick test was used to assess the sensorial response and showed a more rapid recovery of pin prick sensibility in the paw in MME KO. *n* = 11; all tests were analyzed with two‐way repeated measures ANOVA; Sidak's multiple comparison test. Data are presented as mean ± *SEM*; ***p < .001

In order to assess functional consequences of the ablation of MME expression after injury, the motor and sensory behavior of mice was assessed for 1 month following nerve crush. Baseline tests were performed in uninjured animals and no difference between the two groups was observed. Controls and MME KO mice showed a comparable motor function recovery, starting 9 days post injury, as assessed by paw print analysis to determine the sciatic functional index (Figure 7a). Proprioception was assessed by the beam test. As shown in Figure 7b both groups started to recover between Day 9 and Day 13 post injury. As an examination of distal sensorimotor function in these mice we used the toe spreading reflex test; both groups recovered the toe spreading at the same rate following sciatic crush (Figure 7c). Finally, the sensory function was assessed by the pinprick test. MME KO mice showed an increased sensory response compared to control mice with a significantly higher peak already at Day 13 and still at Day 17 after nerve injury. At Day 21, the difference was no longer statistically significant between the groups (Figure 7d). In conclusion, MME ablation does not have any effect on recovery of motor function after nerve crush but the recovery of sensory responses to pinprick stimuli is faster in the MME KO mice.

**Figure 8:**
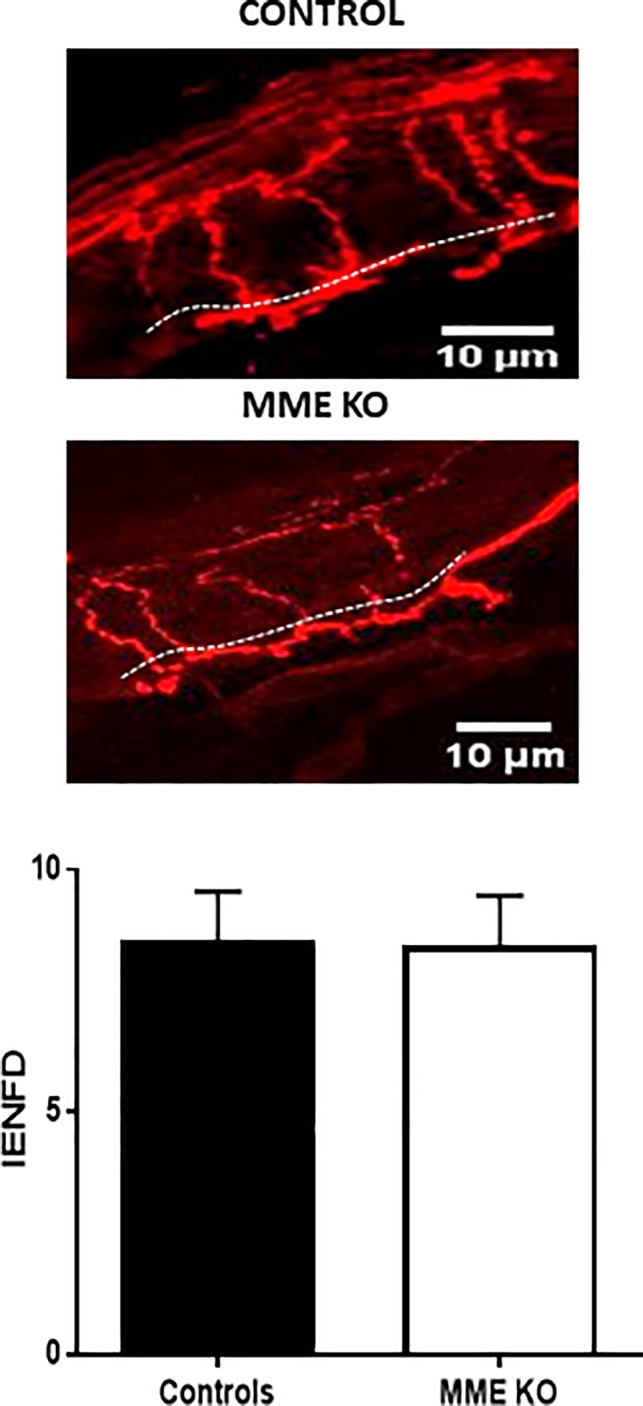
Intra‐epidermal nerve fiber density. Quantification of intra epidermal nerve fibers density in order to assess small fiber reinnervation of the skin at 1 month post crush. This did not show a difference between MME KO and WT controls scale bar 10 μm [Color figure can be viewed at wileyonlinelibrary.com]

Given these behavioral results we investigated cutaneous reinnervation by measuring intraepidermal nerve fiber density (IENFD). One month after injury skin biopsies were collected from MME KO and control mice and stained with the axonal marker PGP9.5. We did not find evidence of altered cutaneous reinnnervation as both groups showed the same IENFD, counted at the microscope as number of fibers crossing the epidermis (Figure 8).

## 4 DISCUSSION

We have investigated the role of MME in nerve repair after injury. The expression of MME is dysregulated after sciatic nerve crush in a NRG1‐dependent fashion. The absence of MME did not, however, result in any difference in the rate of nerve repair at a morphological level and the rate of motor recovery was unchanged. At early phases of recovery enhances responses to pinprick were noted in mice lacking MME, although this may relate to its role in degrading sensitising peptides.

Recently, two articles have reported the association of MME gene mutations with CMT2: the association of bi‐allelic MME mutations with adult‐onset Autosomal Recessive CMT2 in Japanese patients (Higuchi et al., 2016) and also the association of heterozygous MME mutations with late‐onset Autosomal‐Dominant CMT2 in European and American patients (Auer‐Grumbach et al., 2016) raising a possible link between MME expression and axon integrity.

The latter paper reported some changes in the femoral quadriceps nerves of aged mice lacking MME including: more densely packed myelinated fibers as well irregularity and less separation between unmyelinated fibers in Remak bundles. These changes were relatively minor in comparison to the clinical CMT2 phenotype. We did not observe them in the young mice that we studied and hypothesized that if MME has a role in nerve repair we may observe a bigger difference if mice were studied after nerve injury. Effective nerve repair requires the coordinated signaling between multiple cell types (axons, Schwann cells, and immune cells; Taveggia, Feltri, & Wrabetz, 2010). We hypothesized that the loss of MME activity may alter such signaling through its cleavage activity on peptides so impacting on the repair process.

We had noted that MME is a gene which, is expressed in a NRG1 dependent manner following nerve injury (Fricker et al., 2013). NRG1 is a growth factor required during developmental myelination and in the early stage of regeneration after nerve injury. Juxtacrine signaling mediated by the Type III isoforms of NRG1 expressed on regenerating axons determines the rate of remyelination of regenerating axons (Fricker et al., 2011, 2013). Paracrine signaling from Type I NRG isoforms released by Schwann cells also has a role in nerve repair (Stassart et al., 2013). We found that MME expression is downregulated after sciatic nerve crush and increased again about 1 month after injury, a period when remyelination is well established. Interestingly, in absence of NRG1 MME expression is decreased even further and fail to recover at 1 month. These findings are consistent with the previous report that MME was shown to be downregulated in absence of Schwann cell‐axonal contact soon after nerve injury (D'Antonio et al., 2006). The implication is that NRG1 on the surface of axons binds to its cognate receptors (ErbB2/ErbB3) expressed by Schwann cells and determines the expression of MME.

Interestingly in the CNS a similar situation exists in which studying primary hippocampal neurons Xu et al. (2017) demonstrated that NRG1 Type I and III increased the expression of MME.

We considered the possibility of redundancy in the function of MME with other metalloproteases in remyelination. We considered the five reported paralogs of MME (www.genecard.org website): Membrane metallo‐endopeptidase like 1 (MMEL1), endothelin converting enzyme 1 (ECE1), endothelin converting enzyme 2 (ECE2), phosphate regulating endopeptidase homolog X‐linked (PHEX), and endothelin converting enzyme like 1 (ECEL1). None of these paralogs were significantly up or down regulated in our dataset at any time point (dataset of genes in uninjured and injured nerves at Day 10 and Day 28 in conNRG1 mice as reported in (Fricker et al., 2013). Therefore, we could exclude a compensatory mechanism by those paralogs with MME.

Given the link between MME and NRG1 signaling and a potential role of this gene in myelination we examined the expression of MME in the sciatic nerve in the postnatal period at the time of myelination. We found that MME expression increases between P1 and P7 when developmental myelination commences and is maintained at P14, P30, and P60 (Figure 2a,b). We did find that MME expression was significantly decreased in 1 year old WT animals (Figure 2c). The functional significance of this are not known and outside of the scope of this current study examining nerve repair.

We took advantage of the availability of a MME KO mouse model to see whether lack of its expression could affect myelination. We validated this model to show the ablation of MME gene and protein expression in the sciatic nerve (Figure 3). Detailed ultrastructural analysis in 1 month old mice did not demonstrate any difference between MME KO and controls for all the parameters analyzed (i.e., g‐ratio, axon number, and macrophage number. See Figure 4 and Table 1). The processes regulating developmental myelination and remyelination are distinct (Taveggia et al., 2010; Jessen & Mirsky, 2016).

We used the sciatic nerve crush model in adult MME KO and control mice to study both remyelination and axon regeneration. We could not see any morphological difference between the two groups 1 month after the crush with comparable g‐ratio, total axon number, and relative number of myelinated and unmyelinated fibers (Figure 5 and Table 2).

As a final sign of comparable recovery after injury between MME KO and control mice we showed that Schwann cell elongation was not different (Figure 6).

In addition, functional studies revealed a similar rate of motor recovery between the two groups using multiple outcomes including sciatic functional index, toe spreading, and beam walking (Figure 7). The only behavioral difference we found in these MME KO mice was an earlier recovery of pinprick sensibility at 13 days after injury in MME KO mice while controls only recovered at Day 21. This apparently faster recovery in sensorial behavior should, however, been seen in context of the well‐described role that MME has in pain. In a CCI model, MME KO mice showed an increased paw withdrawal response with Von Frey test compared to control mice (Kramer et al., 2009). In this animal model of neuropathic pain there is an elevated inflammatory cascade and the decreased neuropeptide catabolism in MME KO mice resulting in sustained nociceptor excitation. Similarly, in the nerve crush model used here the enhanced sensory responses could relate to sensory neuron sensitization. In support of this we found no difference in the small fibers reinnervation as measured using quantification of IENFD in the paw skin (Figure 8) indicating similar target reinnervation between the two groups at the end of the experiment.

We can therefore conclude that although the expression of MME in the mouse is dysregulated after injury in a NRG1 dependent manner it does not appear to modulate the rate of nerve repair. It does, however, appear to have a role in moderating excessive responses to noxious stimuli. We have not found a correlation to the neuropathy that is reported in patients with MME mutations and this could relate to fundamental differences between rodent and human in the functional role of this enzyme or to the limited lifespan of the mouse when modelling a late onset human neuropathy.

## CONFLICT OF INTEREST

The authors declare no conflict of interest.
